# Preliminary Study on the Effect of Nitrogen Fertilisation on Phytochemical Content Quality of *Gynura procumbens*

**DOI:** 10.21315/tlsr2021.32.3.5

**Published:** 2021-09-30

**Authors:** Mohamad Fhaizal Mohamad Bukhori, Hawa Jaafar, Ali Ghasemzadeh, Uma Rani Sinniah, Gayatri Karipaya

**Affiliations:** 1 Department of Crop Science, Faculty of Agriculture, Universiti Putra Malaysia, 43400 Serdang, Selangor, Malaysia; 2 Centre for Pre-University Studies, Universiti Malaysia Sarawak, 94300 Samarahan, Sarawak, Malaysia; 3 Institute of Biological Sciences, Faculty of Science, Universiti Malaya, 50603 Kuala Lumpur, Malaysia

**Keywords:** *Gynura procumbens*, Herbs, Phytochemical, Nitrogen, Fitokimia, *Gynura procumbens*, Herba, Nitrogen

## Abstract

The study was carried out to examine the effects of nitrogen (N) fertilisation on phytochemical content quality, to determine types of phytochemicals compositions, and to establish phytochemicals marker compounds in *Gynura procumbens*. In this two factors study, three stages of harvesting (H) time and three rates of N were laid out according to Randomised Complete Block Design (RCBD). Physiological and biochemical attributes were recorded to exhibit the trend for plant quality. The results showed that, N has affected phytochemical content significantly (*p* < 0.05) with stronger effect on physiological and biochemical attributes (*p* < 0.01). The results suggested that 0 and 90 kg/ha N, respectively are highly and least effecting the Photo, Cond, TChlC, TCC, TPrc, TLiC, TPC and TFC.

## Abstract (fr)

Kajian ini dijalankan untuk mengkaji kesan baja nitrogen (N) terhadap kualiti kandungan fitokimia, menentukan jenis komposisi fitokimia dan menentukan sebatian penanda fitokimia dalam *Gynura procumbens*. Kajian ini melibatkan dua faktor; masa penuaian dan kadar N, dan mengikut RCBD. Kesan-kesan terhadap faktor fisiologi dan biokimia tumbuhan telah direkodkan dalam tiga peringkat penuaian untuk memperlihatkan corak dan kualiti pertumbuhan dan kandungan fitokimia. Nitrogen memberi kesan yang jelas terhadap kandungan fitokimia (*p* < 0.05), dengan kesan yang signifikan terhadap faktor fisiologi dan biokimia (*p* < 0.01) tumbuhan. Keputusan menunjukkan bahawa 0 dan 90 kg/ha N masing-masing memberi kesan yang sangat tinggi dan paling sedikit terhadap Photo, Cond, TChlC, TCC, TPrC, TliC, TPC dan TFC.

**Highlights**

- Nitrogen fertilisation effecting phytochemical content quality in *Gynura procumbens*.
- Nitrogen fertilisation effecting physiological and biochemical attributes in *G. procumbens*.
- At 90 kg/ha Nitrogen fertilisation, the amount has least effect on photosynthetic rate, stomatal conductance rate, total chlorophyll content, total carbohydrate content, total protein content, total lipid content, total phenolic content, and total flavonoid content in *G. procumbens*.

## INTRODUCTION

As a perennial herbaceous genus of Asteraceae-Senecioneae, *Gynura procumbens* (*G. procumbens*) is among the most important species in Malaysia (Mustaffa *et al*. 2011). In recent years, the interest of plants has increased due to its medicinal potential such as kidney discomfort, rheumatism, diabetes mellitus, constipation and hypertension (Sekar *et al*. 2014). Valuable active biochemical constituent’s percentage has been reported from 1.60% to 13.22% (Morat 2013). The major secondary metabolite components that have been identified from the leaf are flavonoid, phenolic, alkaloids, saponins, anthraquinone glycosides and volatile oils; with the active compounds being, kaempferol-3-*O*-glucoside (7.33%) and phenolic acid (7.20%) (Mou & Dash 2016). The uses of plant-based medicinal compounds are not, however, an insignificant cost. These costs are in part linked to limitations in the quality of plant material. However, these compounds production and accumulation are dependent on the plants performance partly affected by the growing environment (Caretto *et al*. 2015). This includes nutrients supply, which pose as one of the major factors determining productivity in agriculture. The wide ranges of nutrients to be supplied can sometimes cause nutrient deficiency or toxicity effect. Thus, optimum rate of nutrient is important to ensure the growth enhancement and the phytochemical content quality of the plant. Optimum nutrients, especially nitrogen (N), are required for optimal plant growth and development. This is because, N is an essential macronutrient and interactive factor which has significant role as a constituent of chlorophyll and protein synthesis in driving photosynthesis, growth and resource of phytochemical production (Chen *et al*. 2013). Many studies have been reported on the yield affected by nutrient rates in various crops, for example, in *Zingiber officinale* and *Labisia pumila* (Ibrahim *et al*. 2013). However, studies on *G. procumbens* are limited. Therefore, the gap is yet to be established. The general objective of this study is to examine the effects of different rates of N on phytochemical content quality of *G. procumbens*. The specific objectives of this study are to determine the effect of different rates of N on phytochemical and physiological attributes at different harvesting (H) time, to identify the optimum H time for phytochemical yield, and to determine phytochemicals marker compounds.

## MATERIALS AND METHODS

### Experimental Design and Treatments

The study was conducted using randomised complete block design (RCBD) with three blocks and the data was processed using Duncan multiple range test (DMRT) and Pearson’s correlation coefficients, SAS^®^ 9.4 software. The study was a two-factorial experiment. The first factor was four rates of nitrogen (N), *viz*. 0 (0.00 g total per plant) as a control, 30 (0.36 g), 60 (0.72 g) and 90 (1.08 g) kg N/ha (N0, N30, N60 and N90), applied in the form of urea.

The N was split into three fertilisation phases (three months), and each phase (each month) was about 33.3% of total N fertiliser applied at the first week of the month. The second factor was three H time, *viz*. 4, 8 and 12 weeks after treatment. These would give combined treatments of 108. Each combined treatment would give 36 plants per block, giving a total of 108 plants.

### Plant Preparation and Maintenance

The mother plants were obtained from nursery collection in glasshouse and sent for identification in Institute of Bioscience, Universiti Putra Malaysia, voucher specimen (SK 2681/15). The propagation was conducted using cutting from mature stem (10 cm, ≥ 2 internode) and placed in the seed tray containing uniform mixture of sand: coco-peat (1:1 v/v) for rooting purposes (Awang *et al*. 2009). The rooted stem cuttings were then transferred into polyethylene bags (25 cm diameter) containing uniform mixture of soilless medium of coco-peat: burnt paddy husk: well composted chicken manure (5:5:1 v/v/v), and left in glasshouse to acclimatise until ready for treatments (Jaafar *et al*. 2012). The growing media was at pH 5.5 to 7.0. The glasshouse average temperatures were at 22.9°C to 27.2°C with a relative humidity between 50% to 70%. Meanwhile, the average daytime irradiance was at photosynthetic active radiation of 500 μmol m^−2^ s^−1^. The plants were watered manually once a day in the morning or when necessary. If it’s very hot, watering was conducted twice or thrice. Each watering was about 500 mL per plant. Non-chemical pest and disease control was conducted manually at the sight of part presence.

### Sampling

Each combined treatment consisted of 27 plants totalling a sum of 108 plants used in the study. Plants were harvested at weeks 4, 8 and 12 after treatment. Three plants per treatment were sampled at each H time.

### Leaf Gas Exchange

The measurement was obtained from LI-COR^®^ Environmental with closed infrared gas analyser. The operation was automatic, and the data were stored in LI-6400 console and analysed by Photosyn Assistant software. The measurements were carried out between 09:00 a.m. to 11:00 a.m. using fully expanded leaves numbered three and four from plant apex to record photosynthetic carbon assimilation rate (Photo) and stomatal conductance to water rate (Cond). Plants of the different treatments were measured alternately (Ibrahim *et al*. 2014).

### Total Chlorophyll Contents

Total chlorophyll content (TChlC) was measured using fresh weight basis. The third fully expanded leaves from the top of the individual plant were used in the analysis. Leaf disk of 3 mm in diameter was obtained using puncher to acquire 15 consistent sizes per treatment per block. The leaf disks were immediately immersed and incubated in 20 mL of 80% acetone for homogenisation in an aluminum foil-covered glass bottle for approximately 24 h at 5°C until all the green colour had bleached out. Finally, the solution (15 μL) was transferred into 96 well plate to determine absorbances of chlorophyll a (Chl a), chlorophyll b (Chl b) and carotenoids (Car) using uv–vis spectrophotometer at wavelengths of 645, 662 and 470 nm optical density (OD), respectively. Measurements were run in three replicates. The chlorophyll content was calculated as μg g^−1^ fresh weight as per standard equations recommended (Ibrahim *et al*. 2017).


$$\begin{array}{l} \text{Chl a}=[(12.47\times \text{OD }662)-(3.62\times \text{OD }645)\times 10]/(1000\times \text{wt})\\ \text{Chl b}=[(25.06\times \text{OD }645)-(6.50\times \text{OD }662)\times 10]/(1000\times \text{wt})\\ \text{Car}=[(1000\times \text{OD }4700)-(1.29\times \text{Chl a})-(53.78\times \text{Chl b})]/200 \end{array}$$

### Total Carbohydrates Content

Total carbohydrates content (TCC) was measured using Anthrone and Hofreiter’s method (Hansen & Moller 1975). Samples were weighed 1 g into 50 mL conical tube. Sample were hydrolyse by keeping it in boiling water bath for 3 h with 5 mL of 2.5 M hydrochloric acid and cool to room temperature. Then, the sample were neutralise with solid sodium carbonate until the effervescence ceases. Next, the volume was made up to 50 mL and centrifuged at 5,000 rpm for 5 min. The supernatant separated and then filtered with filter paper. The 1 mL of aliquots was taken for analysis. Beforehand, the standards were prepared by taking 0.2, 0.4, 0.6, 0.8 and 1 mL of the working standard. The 0.0 mL serves as blank. The volume was made up to 1 mL in all tubes including the sample tubes by adding distilled water. Cool the contents of all tubes on ice before adding ice-cold anthrone reagent. Then, 4 mL of anthrone reagent were added and heated for 8 min in boiling water bath. The blanks used were absolute methanol. Finally, cool rapidly, then, the solution (15 μL) was transferred into 96 well plate and read the sample absorbance at 630 nm using uv–vis spectrophotometer. Measurements were run in three replicates. The concentration of TCC was calculated according to the following equation that was obtained from the standard glucose graph. The TCC in the sample was expressed as mg glucose equivalent g^−1^ dry sample.


$$\begin{array}{ll} y\; & =0.01x+0.1813\\ R^{2}\; & =0.995 \end{array}$$

All the reagents were prepared fresh prior to use. Anthrone reagent was prepared by dissolving 200 mg of anthrone in 100 mL of ice-cold 95% sulphuric acid. Meanwhile, standard glucose was prepared by preparing the stock of liquid chromatography-grade glucose (G) by dissolving 100 mg in 100 mL of distilled water. The working standard of glucose were prepared by taking 10 mL of glucose stock and diluted to 100 mL with distilled water. The solution was stored refrigerated after adding a few drops of toluene (Ibrahim *et al*. 2017).

### Total Protein Content

Total protein content (TPrC) was measured using Lowry’s method (John 1995). Sample were weight 1 g into 50 mL conical tube and extracted with 10 mL of 100% methanol at room temperature for 24 h and centrifuged at 7,000 rpm for 10 min. The supernatant was separated and then filtered with filter paper. Then, 0.2 mL of extract was pipette out and the volume was made to 1 mL with distilled water. The 5 mL of alkaline copper reagent was added to all tubes and allowed it to stand for 10 min. Then, 0.5 mL of Folin’s Ciocalteau reagent was added and incubated in the dark for 30 min. The blanks used were absolute methanol. Finally, the solution (15 μL) was transferred into 96 well plates to determine the absorbances. The intensity of the colour developed was read at 660 nm using uv–vis spectrophotometer. Measurements were run in three replicates. The concentration of TPrC was calculated according to the following equation that was obtained from the standard bovine serum albumin graph. The TPrC in the sample was expressed as mg bovine serum albumin equivalent g^−1^ dry sample.


$$\begin{array}{ll} y\; & =0.0621x\times 0.1554\\ R^{2}\; & =0.9904 \end{array}$$

All the reagents were prepared fresh prior to use. Standard bovine serum albumin was prepared by preparing the stock of liquid chromatography-grade bovine serum albumin (BSA) by dissolving 20 mg BSA in 10 mL of the same diluents for the samples. Then dilute to 200, 400, 600, 800, 1,000 and 1,200 μg/mL (Ibrahim *et al*. 2011).

### Total Lipid Content

Total lipid content (TLiC) was measured using Folch’s method (Shams *et al*. 2015). Sample were weighed 1 g into 50 mL conical tube and extracted with chloroform and methanol (2:1, v/v) (20 mL) for homogenisation at room temperature for 24 h and centrifuged at 7,000 rpm for 10 min. Lipid extract was purified to eliminate some contaminants by pouring the extracts into a beaker through a filter paper containing activated charcoal to remove coloring matters. A clear supernatant was obtained which was then further purified with the use of 0.2 mL of aqueous 0.9% (w/v) sodium chloride. Purified lipids were transferred into evaporated and concentrated dryness at 40°C, and the residue was weighed. Measurements were run in three replicates. Quantification of crude lipid was performed based on the dry weight determination. The TLiC in the sample was expressed as mg/g dry sample. All the reagents were prepared fresh prior to use (Ibrahim *et al*. 2017).

### Total Phenolic Content

Total phenolic content (TPC) was measured using Folin-Ciocalteu colorimetric assay’s method (Lowry *et al*. 1951). Sample were weight 1 g into 50 mL conical tube and extracted with 10 mL of 100% methanol at room temperature for 24 h and centrifuged at 7,000 rpm for 10 min. The supernatant was separated and then filtered with filter paper. Briefly, 1 mL of extract was pipetted into 15 mL conical tube and 2 mL (10% v/v) of Folin-Ciocalteu reagent was added to the sample and incubated for 5 min. Later, 1.6 mL (7.5%) of sodium carbonate solution was added into the sample. The sample mixture was then vortexed and incubated in the dark for 1 h at room temperature. The blanks used were absolute methanol. Finally, the solution (15 μL) was transferred into 96 well plates to determine the absorbances. Absorbance of the samples was then measured at 760 nm using uv–vis spectrophotometer. Measurements were run in three replicates. The concentration of TPC was calculated according to the following equation that was obtained from the standard caffeic acid graph. The TPC in the sample was expressed as mg caffeic acid equivalents g^−1^ dry sample.


$$\begin{array}{ll} y\; & =0.0098x\times 0.0427\\ R^{2}\; & =0.9942 \end{array}$$

All the reagents were prepared fresh prior to use. Standard caffeic acid was prepared by preparing the stock of liquid chromatography-grade caffeic acid (CA) by dissolving 1 mg in 1 mL of the same diluents for the samples. Then, dilute to 0.1, 0.3, 0.6, 0.9, 1.2, 1.5, 1.8 and 2.0 mg/mL (Ibrahim *et al*. 2017).

### Total Flavonoid Content

Total flavonoid content (TFC) was measured using aluminum chloride complex colorimetric assay’s method (Mongkhonsin *et al*. 2016). Sample were weighed 1 g into 50 mL conical tube and extracted with 10 mL of 100% methanol at room temperature for 24 h and centrifuged at 7,000 rpm for 10 min. The supernatant was separated and then filtered with filter paper. Briefly, 1 mL of extract was pipetted into 15 mL conical tube, mixed with 5 mL of distilled water and 0.3 mL of 5% sodium nitrite solution. Mixed well and the mixture allowed to stand for 6 min and then 0.6 mL of 10% aluminum chloride solution was added. After 5 min, 2 mL of 1M sodium hydroxide was added to the mixture and made up to total volume of 10 mL with distilled water. The blanks used were absolute methanol. Finally, the solution (15 μL) was transferred into 96 well plates to determine the absorbances. Absorbance of the samples was then measured at 510 nm using uv–vis spectrophotometer. Measurements were run in three replicates. The concentration of TFC was calculated according to the following equation that was obtained from the standard kaempferol graph. The TFC in the sample was expressed as mg kaempferol equivalents g^−1^ dry sample.


$$\begin{array}{ll} y\; & =0.0108x\times 0.0435\\ R^{2}\; & =0.9933 \end{array}$$

All the reagents were prepared fresh prior to use. Standard kaempferol was prepared by preparing the stock of liquid chromatography-grade kaempferol (K) by dissolving 1 mg in 1 mL of the same diluents for the samples. Then, dilute to 0.04, 0.20, 0.40, 0.70, 0.90, 1.10, 1.30, 1.50 and 1.80 mg/mL (Kaewseejan *et al*. 2015).

### Preparation of Plant Extract

Sample were weighed 1 g into 50 mL conical tube and extracted with 10 mL of 100% methanol at room temperature for 24 h, sonicated at normal mode for 5 min and centrifuged at 7,000 rpm for 10 min. The supernatant was separated and then filtered with filter paper. The methanolic-extract was transferred into evaporated and concentrated dryness at 40°C using rotary evaporator. It was weighed, redissolved in 1.5 mL liquid chromatography-grade methanol and filtered through sterile membrane filter, 0.45 μm; 25 mm in 2 mL amber glass vials and ready for further chromatographic analysis (Kaewseejan & Siriamornpun 2015).

### Thin Layer Chromatography for TPC and TFC

In thin layer chromatography (TLC) analysis, the stationary phase used was 20 × 20 cm, 0.25 mm plate pre-coated with silica gel 60 F_254_ on aluminum sheets. The mobile phase used was a mixture of toluene, ethyl acetate and formic acid (5:4:1). Marker or reference compounds used were liquid chromatography-grade caffeic acid, cinnamic acid, chlorogenic acid, gallic acid, ferulic acid and vanillic acid for TPC. Meanwhile, kaempferol, quercetin, myricetin and rutin for TFC. Standard compounds were prepared by preparing the stock of compound by dissolving 1 mg in 1 mL of liquid chromatography-grade methanol (Ismail *et al*. 2015). The 10 μL of sample and reference compound were applied as 6 mm band, 2 mm apart, 10 mm from the lower, upper, left, and right edges of the plate using microsyringe. In the glass tank, 50 mL of the developing solvent was poured and allowed to be saturated for 5 to 10 min in room temperature. Migration (as in linear ascending development) distance of the developing solvent on the plate is 80 mm from lower edge of the plate or equivalent to the time allowed for the development and maximal separation of the active compounds present in the samples was 15 to 25 min. The plates were then dried at 100°C using oven for 3 to 5 min. Dried plates were visualised by observing under uv light at 254 nm and 366 nm. Spot detection was done qualitatively. The colour and distance of the unknown spots were compared with reference compound. The retardation factor (*R_f_*) values were calculated using the formula of migration distance of spot/migration distance of solvent (Ismail *et al*. 2015).

### High-Performance Liquid Chromatography (HPLC) for TPC

The HPLC-grade purity chemicals and reference compounds such as methanol, acetonitrile, orthophosphoric acid 85%, caffeic acid, cinnamic acid, chlorogenic acid, gallic acid, ferulic acid and vanillic acid were used in the analysis. The linear standard curves were constructed by injecting range of 0.05, 0.07, 0.09, 0.11, 0.13, 0.15 and 0.17 mg of caffeic acid mL^−1^ of methanol. Filtered water was obtained from ultrapure water purification system. All the reference compound stock solutions were prepared by dissolving 1 mg in 1 mL of liquid chromatography-grade methanol, filtered through sterile membrane filter, 0.45 μm; 25 mm in 2 mL amber glass vials, and stored at 5°C. Diluted working solutions were freshly prepared for each analysis (Giedyk 2011). The system was equipped with Waters^®^ 600E multisolvent delivery system, USA consisting of UV/Visible detector (Waters^®^ 2489), degasser (Waters^®^), autosampler (Waters^®^ 2707) and Waters Empower 3 software. Separation was achieved on reversed phase column [RP-18e (100 × 4.6 mm i.d; 2 μm), semiprep, particle size 0 μm, pore size 130 Å, Chromolith^®^] preceded by C18 guard column at 40°C with diode array detector (DAD) set at 190 to 600 nm and (0.1%) orthophosphoric acid 85% (eluent A): acetonitrile (eluent B) (90.5:9.5, v/v isocratically) employed as the eluent. Column was washed with 100% methanol for 10 min, the initial conditions were again applied, the system was monitored for 30 min and the column allowed to equilibrate with (0.1%) orthophosphoric acid 85%: acetonitrile (90.5:9.5, v/v isocratically) for 35 min, being 75 min the total consuming time of run. The temperature of the oven was 40°C. The flow rate was kept constant at 0.7 mL min^−1^ and the peaks were simultaneously identified using uv absorbance at 327 nm for caffeic acid. The injection volume was 1 μL (standard compound) and 10 μL (sample) for each technical repeat automatically. Quantification was carried out by integration of the peak using external standard method. The procedure was performed separately for each standard (Yi *et al*. 2016). This method was linear in the range tested and had high linear regression coefficient of *r* = 0.9936 between concentrations and peak areas.

### HPLC for TFC

The HPLC-grade purity chemicals and reference compounds such as methanol, acetonitrile, orthophosphoric acid 85%, kaempferol, quercetin, myricetin and rutin were used in the analysis. The linear standard curves were constructed by injecting range of 0.2, 0.3, 0.4, 0.5 and 0.6 mg of kaempferol mL^−1^ of methanol. Filtered water was obtained from ultrapure water purification system. All the reference compounds stock solutions were prepared by dissolving 1 mg in 1 mL of liquid chromatography-grade methanol, filtered through sterile membrane filter, 0.45 μm; 25 mm in 2 mL amber glass vials, and stored at 5°C. Diluted working solutions were freshly prepared for each analysis (Giedyk 2011). The system was equipped with Waters^®^ 600E multisolvent delivery system, USA consisting of UV/Visible detector (Waters^®^ 2489), degasser (Waters^®^), autosampler (Waters^®^ 2707) and Waters Empower 3 software. Separation was achieved on reversed phase column [RP-18e (100 × 4.6 mm i.d; 2 μm), semiprep, particle size 0 μm, pore size 130 Å, Chromolith^®^] preceded by C18 guard column at 40°C with diode array detector (DAD) set at 190 to 600 nm and (0.03M) orthophosphoric acid 85% (eluent A): methanol (eluent B) (60:40, v/v isocratically) employed as the eluent. Column was washed with 100% methanol for 10 min, the initial conditions were again applied, the system was monitored for 30 min and the column allowed equilibrating with orthophosphoric acid 85%: methanol (60:40, v/v isocratically) for 35 min, being 75 min the total consuming time of run. The temperature of the oven was 35°C. The flow rate was kept constant at 1 mL/min and the peaks were simultaneously identified using uv absorbance at 250 to 360 nm for kaempferol. The injection volume was 10 μL (standard compound and sample) for each technical repeat automatically. Quantification was carried out by integration of the peak using external standard method. The procedure was performed separately for each standard (Shoeva *et al*. 2016). This method was linear in the range tested and had high linear regression coefficient of *r* = 0.9953 between concentrations and peak areas.

### Statistical Analysis

The data were subjected to analysis of variance (ANOVA) and correlation using SAS^®^ 9.4 software (Version 8.0, SAS Institute Inc., and Cary, NC, USA). The analysis was done in triplicate and expressed as mean (*n* = 3) ± standard error (SE) from the dependent treatments (Jaafar *et al*. 2012). The variables from measurements were analysed using General Linear Model with N supply managements. Any differences between treatment means were analysed by two-way analysis and compared using DMRT at *p*-value < 0.05 levels. The regression model that best fitted the data, evaluated by an *F*-test, was chosen.

## RESULTS

### Leaf Gas Exchange

**Figure 1:**
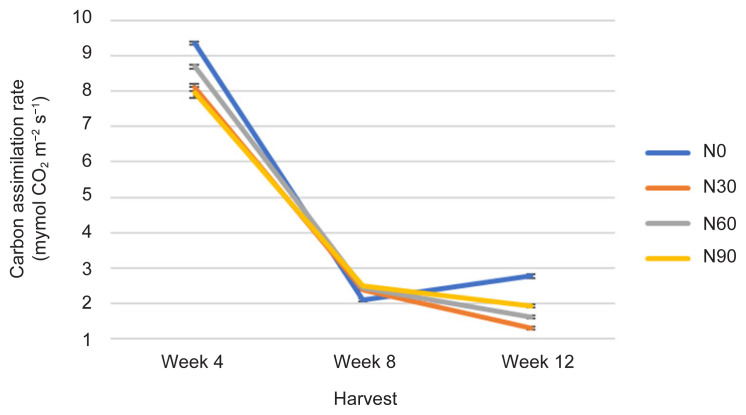
Changes in Photo as affected by N rate and H time.

Photosynthetic rate was affected in all treatment including the control treatment (N0), (*p* ≤ 0.05) (Fig. 1, Tables 1 and 2). The Photo in N0 was highest at week 4. As treatment continued to week 8, Photo has decreased lower than other treatments. Continuing the treatment to week 12, the N0 recorded the highest Photo than other treatment at almost 3 μmol CO_2_ m^−2^ s^−1^. The Photo in N90 was higher than N30 and N60 at week 12. The N30 has t h e lowest Photo compared to N0. Photo was highest in N0 at week 4 when plants are young and this continue until week 12 followed by N90, N60 and N30. This is because Photo decreased with continuing H time and increasing N rate. Plants in N0 displayed highest Photo (8.846 μmol CO_2_ m^−2^ s^−1^) at week 4 followed by N60 (8.184 μmol CO_2_ m^−2^ s^−1^), N30 (7.601 μmol CO_2_ m^−2^ s^−1^) and N90 (7.454 μmol CO_2_ m^−2^ s^−1^). As plant aged at weeks 8 and 12, Photo decreased especially at week 12, following this order: N0, N90, N60 and N30 with respective values of 0.0510 μmol CO_2_ m^−2^ s^−1^, 0.0484 μmol CO_2_ m^−2^ s^−1^, 0.0445 μmol CO_2_ m^−2^ s^−1^ and 0.0407 μmol CO_2_ m^−2^ s^−1^ (Fig. 1). The event was because the plants in N0 had enough N from the growth media for higher photosynthetic capacity and light utilisation. Suggesting N0 is highly respond to nutrient acquisition and significantly affected by water availability and carbon use efficiency (Wang *et al*. 2016). Measurements of Photo are significantly correlated with Cond, TPDW, NoL and TLA at *r* = 0.860; *p* ≤ 0.0001, *r* = −0.357; *p* ≤ 0.05, *r* = −0.459; *p* ≤ 0.01 and *r* = −0.489; *p* ≤ 0.01, respectively by a linear function (Table 3).

**Figure 2:**
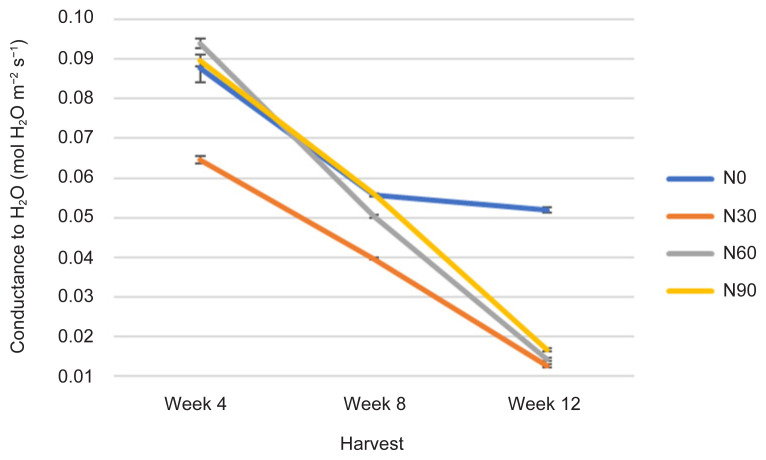
Changes in Cond as affected by N rate and H time.

Stomatal conductance was affected in all treatments including the control treatment (N0) and decreased with increasing age indicated by H time (*p* ≤ 0.05) (Fig. 2, Tables 1 and 2). The Cond of plants in N60 recorded highest value at week 4 followed by N90, N0 and lowest value in N30. As plant aged, Cond in N60 was lower than N90 but like N30. Instead, Cond N0 was higher than other treatments at longer H time (week 12). The N30 has lowest Cond compared to N0. Stomatal conductance was highest in N60 at Week 4 compared to N30 at Week 12. The Cond seemed to decrease with increasing N rates especially with increasing H time because of pre-adaptation, adaptation and post-adaptation during plant growth (Heydarizadeh 2016). In the early stage of growth, stomatal are in pre-adaptation during the acclimatisation, therefore Cond was low, following adaptation during the continuous treatment, therefore, Cond was increased, and finally post-adaptation where Cond was decreased during the growth terminal (Wuyts *et al*. 2015). The Cond was the highest in N60 at week 4, and lowest in other treatments especially when plant aged at week 12 (Fig. 2). Plants in N60 had the lowest intercellular CO_2_ concentration (232 mol H_2_O m^−2^ s^−1^) compared to other treatments (N90, 244 mol H_2_O m^−2^ s^−1^; N0, 296 mol H_2_O m^−2^ s^−1^; and N0, 302 mol H_2_O m^−2^ s^−1^), which represented higher carbon assimilation and could have directly contributed to active Cond (0.094 mol H_2_O m^−2^s^−1^) (Fig. 1). Meanwhile, plants in N30 were perhaps experiencing diffusion resistance along the study. This finding suggested t h a t N0 is highly stimulated on stomatal opening and closing (Selmar & Kleinwächter 2013). Measurements of Cond are significantly correlated with Photo at *r* = 0.860; *p* ≤ 0.0001 by a linear function (Table 3).

### Total Chlorophyll Contents

**Figure 3:**
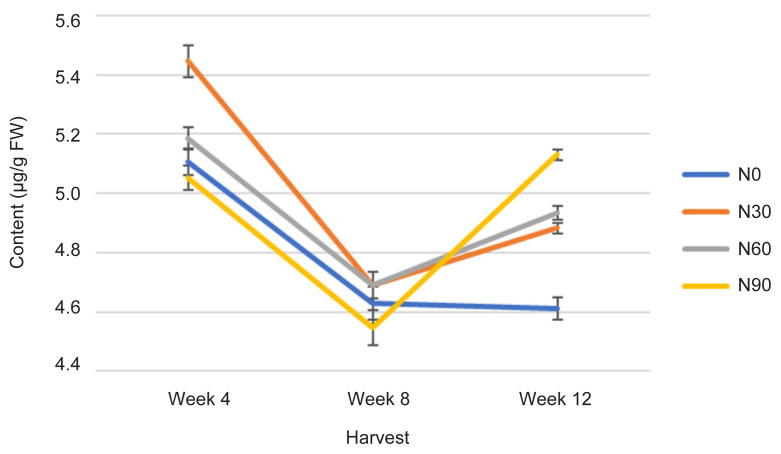
Changes in TChlC as affected N rate and H time.

The TChlC was statistically affected with increasing rate of N and age as indicated by the H time (*p* ≤ 0.05) (Fig. 3). At week 4, the N30 treatment recorded highest value of TChlC compared to other treatments which were not significantly different to each other with the lowest value recorded in N90. However, as the plant aged to weeks 8 and 12, the N90 recorded increased TChlC (5.18 μg/g FW); followed by N60 (4.90 μg/g FW), N30 (4.88 μg/g FW) and N0 (4.60 μg/g FW). The TChlC was the highest in N30 at week 4 compared to N0 at week 12 (Fig. 3). The values are concurrent to Photo and Cond rates at week 4 (Figs. 1 and 2), where gas exchange rate leaf was high, and decreased during week 8 and subsequently increased by 12% at week 12. The event occurred because of pre-adaptation (acclimatisation), adaptation (growth) and post-adaptation (maturing) during plant growth cycle where N is vital component of plants energy metabolism and of activated intermediates in the photosynthetic and primary metabolism following the growth cycle requirement accordingly (Pant *et al*. 2015). Meanwhile, high N rate reported significantly decreased chlorophyll content regardless of any types of growth media (Rahimi *et al*. 2013). Sufficient rates of N in N30 was determined the TChlC highest in the treatment where the content was maintained at a rate which was dependent on the rate of N uptake proportionally with Photo and Cond rates (Mohd Zaidan *et al*. 2014). Measurements of TChlC are significantly correlated with Photo at *r* = 0.674; *p* ≤ 0.0001 by a linear function (Table 3).

### Phytochemical Content Quality

#### Total carbohydrates content (TCC)

**Figure 4:**
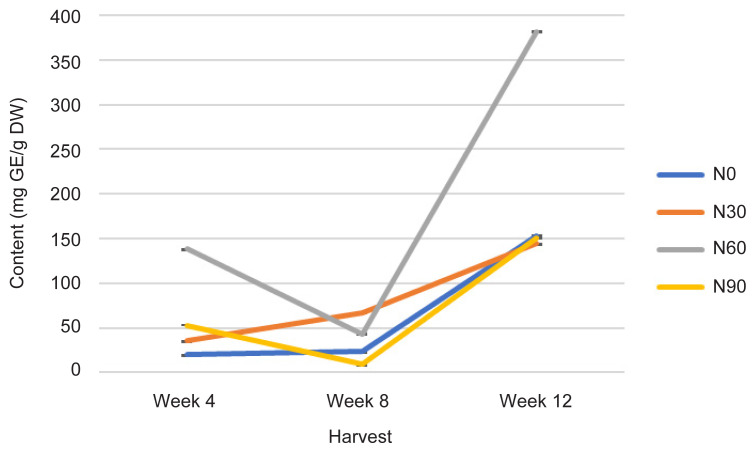
Changes in TCC as affected by N rate and H time.

The TCC was statistically affected with increasing rate of N at different plant age as represented by the H time (*p* ≤ 0.05) (Fig. 4, Tables 1 and 2). The N60 treatment has increased tremendously TCC followed by N90, N30 and N0 (control treatment) when applied during early growth at week 4. As the growth progressed to week 12, N60 continued to increase TCC from 138 to 381 mg GE/g DW compared to N90, which value did not continue to surpass all other treatments; instead, the value was almost like all other N treatments (Fig. 4). Nitrogen is an essential element constituent of plant compounds in plant growth (Rostami *et al*. 2012). The TCC was increased significantly with continuing H time and increasing N rates (Hassan & Ali 2014). The event occured because of N would increase sugar content of plants (Aminifard *et al*. 2012). Sugar content play an important role in plants osmotic adjustment and may protect against oxidative stress, which also suggesting N input could not alter organic carbon allocation such as carbohydrate (Rahimi *et al*. 2013). Measurements of TCC are significantly correlated with Photo, TPrC, TLiC, TPC and TFC at *r* = −0.340; *p* ≤ 0.05, *r* = 0.542; *p* ≤ 0.001, *r* = 0.379; *p* ≤ 0.05, *r* = 0.600; *p* ≤ 0.0001 and *r* = 0.367; *p* ≤ 0.05, respectively by a linear function (Table 3).

#### Total protein content (TPrC)

**Figure 5:**
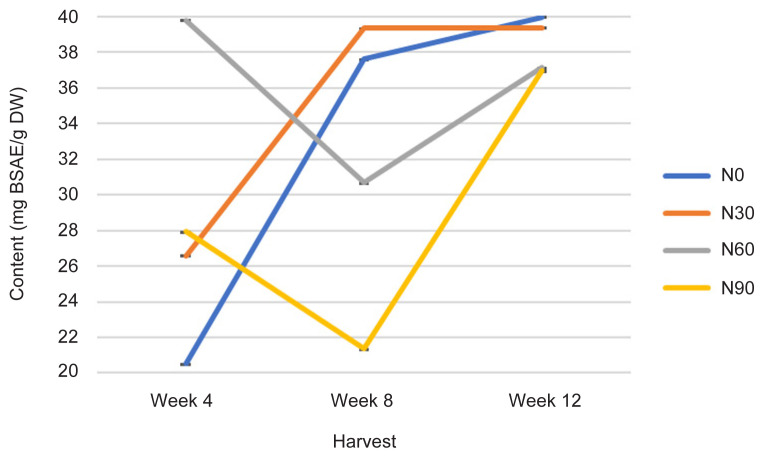
Changes in TPrC as affected by N rate and H time.

The TPrC was statistically affected with increasing rates of N and H time (*p* ≤ 0.05) (Fig. 5, Tables 1 and 2). At early growth stage (week 4), the N60 treatment recorded highest TPrC value (39 mg BSAE/g DW) followed by N90 (28 mg BSAE/g DW), N30 (26 mg BSAE/g DW) and N0 (control treatment) (20 mg BSAE/g DW). However, during week 12, TPrC has decreased in N60, which showed lower and almost similar with N90 value. Meanwhile, TPrC has increased tremendously in N0 and N30 with increasing H time from week 8 to 12. The TPrC was highest in N0 and N30, and lowest in N60 and N90 with continuing H time (Fig. 5). The event was because of plants protein contents in each treatment are affected by N rates. Increasing N rate increased protein content, N uptake and decreased N use efficiency. However, the effects are dependent on plant moisture supply as well (Khalid 2006). Meanwhile, in N0 and N30 sufficient N rates lead to effective photosynthesis rates by increasing the proportion of soluble protein (Chen *et al*. 2013). At early growth stage higher N rate has higher TPrC but as the plant aged, lower N rate provided higher TPrC could be related to value recorded in Photo (Fig. 1) as well as total biomass, number of leaves and total leaf area presented by N60 and N90. Nitrogen plays a crucial role in determining photosynthetic productivity in both natural and agricultural environment (An & Shangguan 2008). This is because, N is used by plants to build many organic compounds: amino acids, proteins, enzymes, and nucleic acids (Pant *et al*. 2015). Measurements of TPrC are significantly correlated with Photo, TCC, TLiC, TPC and TFC at *r* = −0.478; *p* ≤ 0.01, *r* = 0.542; *p* ≤ 0.001, *r* = 0.540; *p* ≤ 0.001, *r* = 0.935; *p* ≤ 0.0001 and *r* = 0.632; p ≤ 0.0001, respectively by a linear function (Table 3).

#### Total lipid content (TLiC)

**Figure 6:**
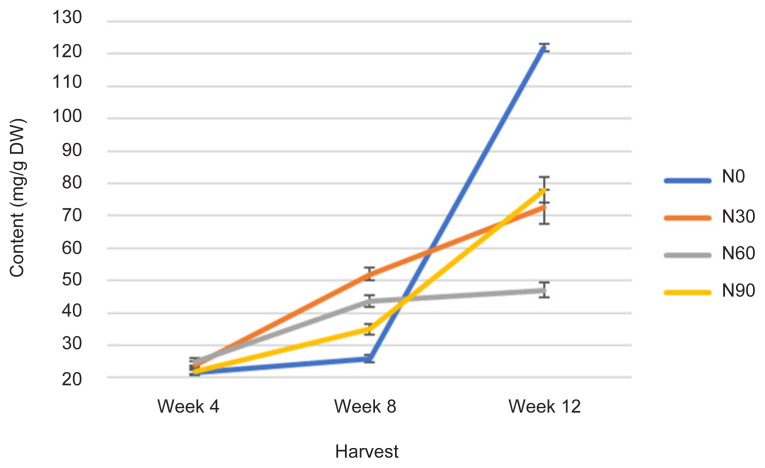
Changes in TLiC as affected N rate and H time.

The TLiC was statistically affected with increasing rates of N and H time (*p* ≤ 0.05) (Fig. 6, Tables 1 and 2). At early growth, all N treatments did not influence the TLiC. However, with increase in age from week 8 to week 12, N0 (control treatment) has increased the value of TLiC tremendously from the lowest value at 25 mg/g DW to 120 mg/g DW. Meanwhile, N30 which recorded highest value at week 8 (50 mg/g DW) compared to all the N treatments, upon reaching week 12 has recorded lower TLiC than that recorded by N0 but equal to N90 and higher than TLiC value in N60 (Fig. 6). The TLiC has significantly increased with continuing H time and rising N rates. The event were because of metabolically, synthesis in lipid content may be related to development of intracellular membranous compartmentation and the availability of intracellular (non-plasmic and periplasmic) spaces as plant ages (Ora & Anekwe 2013). Since vacuoles are intracellular spaces function as accumulation sites for plant metabolites, therefore, vacuolar membranes might play a pivotal role in compartmentalised acquisition, sequestration and retention of metabolites, including lipids (Mishra *et al*. 2006). On the other hands, TLiC has increased during leaf expansion in order to attain optimum level at full maturity and substantial decline in senescing tissue of old plants (Mishra *et al*. 2006). In this event, N is an essential element of key macromolecules such as proteins, nucleic acids, some lipids, and chlorophylls (Galieni *et al*. 2015). Measurements of TLiC are significantly correlated with Photo, TCC, TPrC, TPC and TFC at *r* = −0.563; *p* ≤ 0.001, *r* = 0.379; *p* ≤ 0.05, *r* = 0.540; *p* ≤ 0.001, *r* = 0.667; *p* ≤ 0.0001 and *r* = 0.776; *p* ≤ 0.0001, respectively by a linear function (Table 3).

#### Total phenolic content (TPC)

**Figure 7:**
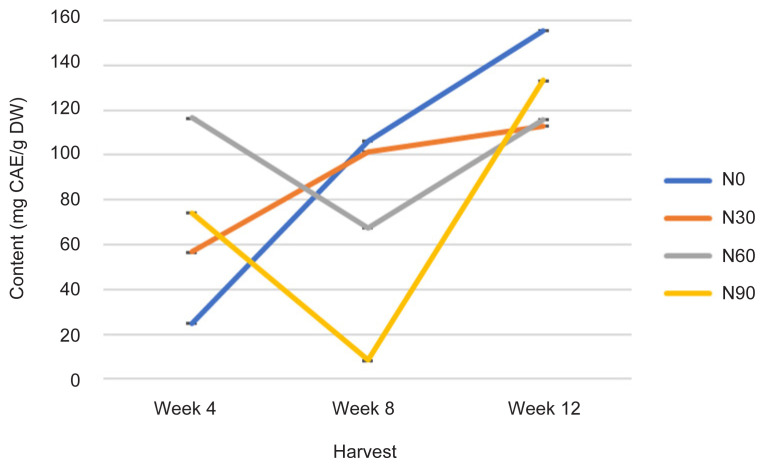
Changes in TPC as affected by N rate and H time.

The TPC was statistically affected with increasing rates of N at different H time (*p* ≤ 0.05) (Fig. 7, Tables 1 and 2). At week 4, TPC value was significantly decreased with different N treatment in descending manner N60 (116 mg CAE/g DW) >N90 (74 mg CAE/g DW) > N30 (56 mg CAE/g DW) > N0 (24 mg CAE/g DW). As the plant aged indicated by H time from week 4 to week 12, TPC has increased tremendously in N0 (control treatment) followed by N90, N60 and N30 (Fig. 7). The event could be due to low N supply has allowed an increase in phenolic content and following low supply also provide continuous increments in phenolics contents (Ibrahim *et al*. 2013). The increased of phenolic contents in plant tissues was due to low N availability (N0 and N30); however, over long H time, this treatment will have a negative effect on plant growth. The low N supply over long period resulted in an up-regulation of phenolic biosynthetic pathway, as demonstrated by an increase in TPC (Anulika *et al*. 2016). Measurements of TPC are significantly correlated with Photo, TCC, TPrC, TLiC and TFC at *r* = −0.394; *p* ≤ 0.05, *r* = 0.600; *p* ≤ 0.0001, *r* = 0.935; *p* ≤ 0.0001, *r* = 0.667; *p* ≤ 0.0001 and *r* = 0.653; *p* ≤ 0.0001, respectively by a linear function (Table 3).

#### Total flavonoid content (TFC)

**Figure 8:**
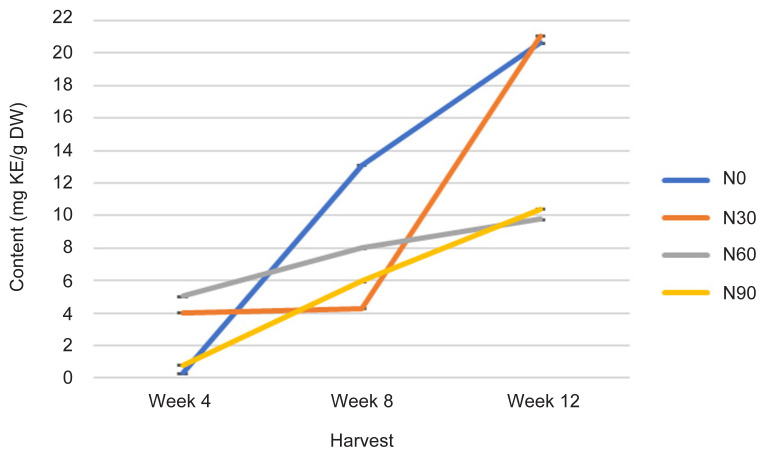
Changes in TFC as affected by N rate and H time.

The TFC was statistically affected with increasing rates of N at different H time (*p* ≤ 0.05) (Fig. 8, Tables 1 and 2). At week 4, TFC value was significantly decreased with different N treatment in descending manner N60 (5 mg KE/g DW) >N30 (4 mg KE/g DW) > N90 (1 mg KE/g DW) > N0 (0.2 mg KE/g DW). As the plant aged indicated by H time from week 4 to week 12, TFC has increased tremendously in N0 (control treatment) followed by N30, N90 and N60 (Fig. 8). The event occured because of N availability has implicated phytochemical response in plants (Galieni *et al*. 2015), where N deficiency induces the content of flavonoids and flavonols (Pal *et al*. 2015). High N supply (N60 and N90) increases the content of nitrogenous compounds, such as free amino acids (protein) (Fig. 5), which however, reduce the secondary metabolites content such as flavonoid (Larbat *et al*. 2012). On the other hands, N enhanced plant N content and biomass production, however, further increases in N rate had no influence even declined the secondary metabolites content (Davies *et al*. 2009). Measurements of TFC are significantly correlated with Photo, TCC, TPrC, TLiC and TPC at *r* = −0.677; *p* ≤ 0.0001, *r* = 0.367; *p* ≤ 0.05, *r* = 0.632; *p* ≤ 0.0001, *r* = 0.776; *p* ≤ 0.0001 and *r* = 0.653; *p* ≤ 0.0001, respectively by a linear function (Table 3).

### Phytochemical Marker and Composition Quality

#### Thin layer chromatography

**Figure 9:**
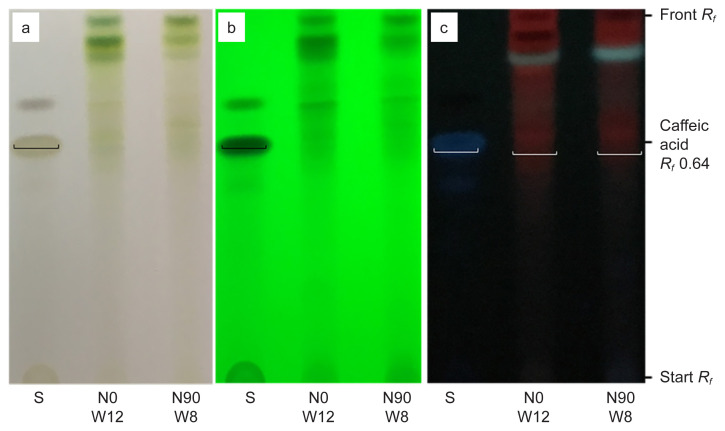
TLC developed-profiles of caffeic acid (S) of methanolic extract of dried leaves powder for N0 W12 (control treatment, week 12) and N90 W8 (90 kg N/ha treatment, week 8) observed under visible light, (a) 254 nm, (b) and 366 nm and (c) before derivatisation.

For phenolic compound, qualitative analysis of methanol extract using TLC was performed using a mixture of toluene: ethyl acetate: formic acid (5:4:1 v/v) used as the mobile phase (Sasidharan *et al*. 2011) yielded a good resolution of caffeic acid with bands at *R_f_* = 0.64 (Fig. 9). Caffeic acid were identified as phytochemical marker in phenolic compounds analysis and quantified at visible light and visible wavelength of 254 and 366 nm before derivatisation (Fig. 9). The results revealed that methanol extract at 1 g/10 mL of 100% methanol contained caffeic acid in accordance to TPC analysis, where highest in N0 (control treatments) at week 12 (158 mg CAE/g DW), and lowest in N90 at week 8 (10 mg CAE/g DW) (Fig. 7). This TLC procedure can be used as a fast-screening method for *G. procumbens* leaf samples and herbal formulations.

**Figure 10:**
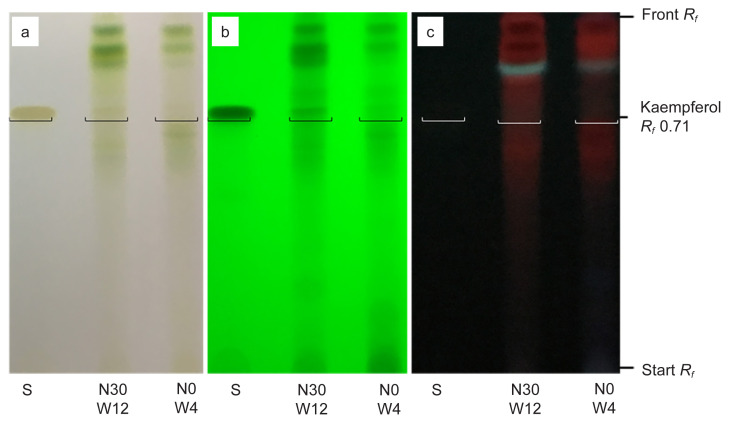
TLC developed-profiles of kaempferol (S) of methanolic extract of dried leaves powder for N30 W12 (30 kg N/ha treatment, week 12) and N0 W4 (control treatment, week 4) observed under visible light, (a) 254 nm, (b) and 366 nm and (c) before derivatisation.

For flavonoid compound, qualitative analysis of methanol extract using TLC was performed using a mixture of toluene: ethyl acetate: formic acid (5:4:1 v/v) used as the mobile phase (Sasidharan *et al*. 2011) yielded a good resolution of kaempferol with bands at *R_f_* = 0.71 (Fig. 10). Kaempferol were identified as phytochemical marker in flavonoid compounds analysis and quantified at visible light and visible wavelength of 254 and 366 nm before derivatisation (Fig. 10). The results revealed that methanol extract at 1 g/10 mL of 100% methanol contained kaempferol in accordance with TFC analysis, where highest in N30 at week 12 (21 mg KE/g DW), and lowest in N0 (control treatment) at Week 4 (0.2 mg KE/g DW) (Fig. 8). This TLC procedure can be used as a fast-screening method for *G. procumbens* leaf samples and herbal formulations.

#### High-performance liquid chromatography

**Figure 11:**
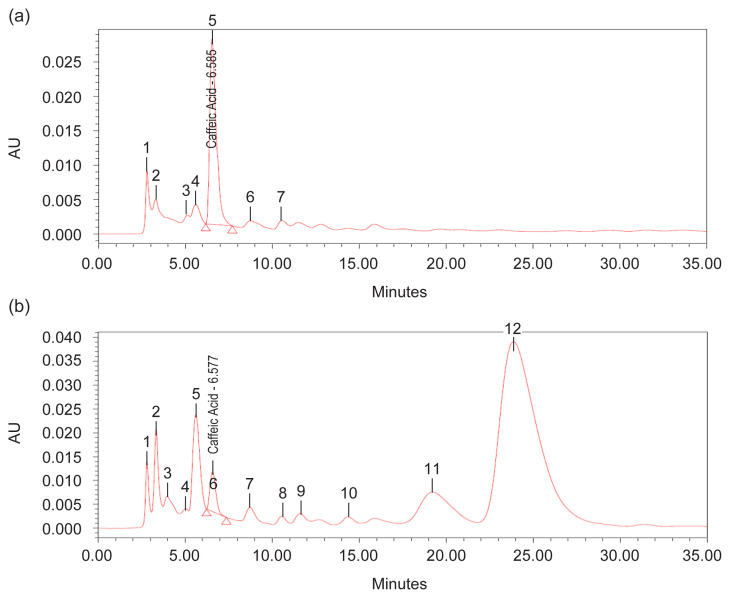
HPLC developed-profiles of caffeic acid standard solution (1.0 mg/mL) at *t_r_* = 6.5 min of methanolic extract of dried leaves powder for (a) N0 treatment week 12 and (b) N90 treatment week 8 observed under 327 nm derivatisation.

For phenolic compound, quantitative analysis of methanol extract using HPLC was performed using a mixture of 0.1% of 85% orthophosphoric acid: acetonitrile (90.5:9.5, v/v isocratically) (Kaewseejan & Siriamornpun 2015) used as the eluent yielded a good peak of caffeic acid at *t_r_* = 6.5 min, 327 nm (Fig. 11). The results revealed that methanol extract at 1 g/10 mL of 100% methanol contained caffeic acid as phytochemical marker in accordance with TPC analysis, where highest in N0 (control treatments) at week 12 (158 mg CAE/g DW), peak 5, *t_r_* = 6.585 min, 56.38% area, and lowest in N90 at week 8 (10 mg CAE/g DW), peak 6, *t_r_* = 6.577 min, 3.12% area) (Figs. 7 and 9). This HPLC procedure can be used as a fast screening method for *G. procumbens* leaf samples and herbal formulations.

**Figure 12:**
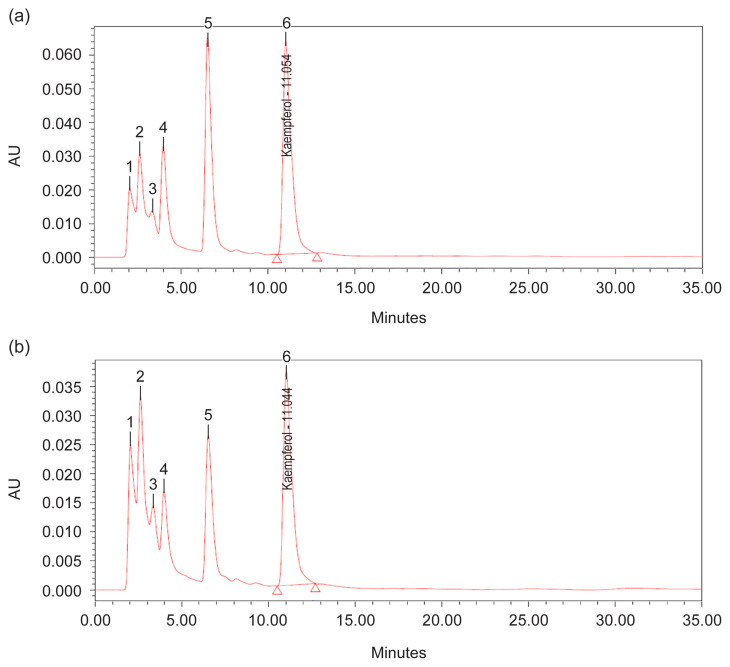
HPLC developed-profiles of kaempferol standard solution (1.0 mg/mL) at *t_r_* = 11.0 min of methanolic extract of dried leaves powder for (a) N30 treatment week 12 and (b) N0 (control treatment) week 4 observed under 250 to 360 nm derivatisation.

For flavonoid compound, quantitative analysis of methanol extract using HPLC was performed using a mixture of 0.03M of 85% orthophosphoric acid: methanol (60:40, v/v isocratically) (Yi *et al*. 2016) used as the eluent yielded a good peak of kaempferol at *t_r_* = 11.0 min, 250 to 360 nm (Fig. 12). The results revealed that methanol extract at 1 g/10 mL of 100% methanol contained kaempferol as phytochemical marker in accordance to TFC analysis, where highest in N30 at week 12, peak 6, *t_r_* = 11.054 min, 14.40% area, and lowest in N0 (control treatment) at week 4, peak 6, *t_r_* = 11.044 min, 10.33% area (Figs. 8 and 10). This HPLC procedure can be used as a fast-screening method for *G. procumbens* leaf samples and herbal formulations.

## DISCUSSION

All physiological (Photo, Cond and TChlC) and biochemical traits (TCC, TPrC, TLiC, TPC and TFC) were markedly influenced by N rates and H time, and the interactions between these two factors (Figs. 1–8). Growth-differentiation balance and carbon-nutrient balance hypothesis indicated that N affected physiological traits and phytochemical content (primary and secondary metabolite synthesis) (Kleczewski *et al*. 2010). Based on harvest index analysis, N0 (control treatment) and N90 represent the highest and least effect respectively on *G. procumbens* physiology traits and phytochemical content. The Photo and Cond have decreased with increasing N rate (Figs. 1 and 2). However, in order to maximise the plant biomass, the plant requires optimisation of Photo and Cond via regulation of N rate supply (Ali *et al*. 2012). Meanwhile, maximising phytochemical content are also associated with the environment, in this case plant’s interaction with N availability and plant age (H time) (Ismail *et al*. 2017). Therefore, any agronomic factors which influence plant growth and development may also influence phytochemical content (primary and secondary metabolites). It is appropriate to examine the effects of N (interactive factor over time) in driving photosynthesis and resource to phytochemical content (Galieni *et al*. 2015). This is because, N influence physiological traits and secondary metabolism through its role in regulating primary metabolite synthesis (Wan *et al*. 2015). Consequently, it is important to maximise physiological traits (Photo and Cond) over primary (TCC, TPrC and TLiC) and secondary metabolite (TPC and TFC) content to achieve optimal yield per plant of the active component.

Phytochemical content was driven by high photosynthetic rate (An & Shangguan 2008). This is because, sufficient N in the plant will complete a chlorophyll constituent and protein synthesis especially when plants have grown older (Galieni *et al*. 2015). And this protein synthesis originally from amino acids are a precursors of secondary metabolites formation (flavonoids) (Hew *et al*. 2013). In this study, increased Photo (Fig. 1) especially under N0 (control treatment) and N60 subsequently resulted in increased most of the primary and secondary metabolites over H time. The primary and secondary metabolites values were highest at low N application (N0 and N30) especially during early growth stage (Figs. 4, 5, 6, 7 and 8). As N rate increased in N60 and N90, metabolite content decreased significantly (*p* ≤ 0.05) (Figs. 4–8). This indicates the increase of N rate, decreases the Photo and Cond, therefore, stimulates primary and secondary metabolite synthesis significantly (Galieni *et al*. 2015). As reported before, the interlinking of carbon and N metabolism beyond the requirements for growth and development are well known, therefore, for carbon-and N-based metabolites synthesis in plant growth the carbon fluxes between growth and metabolite synthesis in preparing for low N rates available in the plants (Caretto *et al*. 2015). The event also could be during early stage of growth, plants have already fractioned the N throughout the plant tissues or there is no more N requires for growth, thus, available sources of N was converted to secondary metabolites (Pal *et al*. 2015).

The effect of N on physiological traits and phytochemical content have provides insight of possible mechanism, in which pattern of photosynthetic rate against metabolite content in *G. procumbens*. Therefore, single N treatment may not be the only explanation of the observed plants effects and N tissue status of plants especially early growth is also required (Larbat *et al*. 2012). However, this study can only speculate how the plant growth and metabolite content including biomass, TCC, TPrC, TLiC, TPC and TFC are linked to N supplies. And also, N role in chlorophyll content, the primary light harvesting compound of photosynthesis (An & Shangguan 2008), where, in this study, TChlC was statistically affected with increasing N rate and H time (*p* ≤ 0.05) (Fig. 3). The TChlC values are concurrent to Photo and Cond rates at week 4 (Figs. 1 and 2), where gas exchange rate leaf was high, and decreased during Week 8 and subsequently increased at week 12. Meanwhile, qualitative, and quantitative analysis developed using TLC and HPLC respectively has markedly caffeic acid and kaempferol as phytochemical markers for phenolic and flavonoid content measurements.

## CONCLUSION

Nitrogen affected phytochemical content significantly (*p* < 0.05) with higher effect on physiological traits and biochemical attributes (*p* < 0.01). Harvest index analysis of secondary metabolite content have shown the effect of N treatment in the following descending manner, N0 (control treatment)>N30>N60>N90 as explained in growth-differentiation balance and carbon-nutrient balance hypothesis. The results suggest that N0 and N90 were high and least, respectively, effective for enhancing physiological traits (Photo, Cond and TChlC); and phytochemical content (TCC, TPrC, TLiC, TPC and TFC). The results have shown N treatment especially when interacting with H time representing plant age are highly significant (*p* < 0.0001) in physiology and phytochemical effect. These effects because of N are required in copious quantities and must be in balance with other fertiliser to achieve its maximum yield potential. However, surplus N was observed to decrease phytochemical contents especially when plants grow older. The results also show that Photo is significantly correlated (*p* ≤ 0.05) with Cond, TChlC, TCC, TPr.C, TLiC, TPC and TFC. Thus, high plant growth and metabolites content of *G. procumbens* should be improved through the selection of appropriate N rates, proper fertiliser interaction, and plant age at which high total metabolite production can be achieved.

## Figures and Tables

**Table 1:** Probability of greater *F* (*P* > *F*) for the ANOVA on effect of N rates and H times on growth, physiology and biochemical variables.

Source	N	H	N × H	CoV
Df	3	2	6	
Photo	****	****	****	2.866
Cond	****	****	****	3.638
TChlC	****	****	****	0.609
TCC	****	****	****	0.020
TPrC	****	****	****	0.027
TLiC	****	****	****	5.057
TPC	****	****	****	0.003
TFC	****	****	****	0.226

*Notes*: All analyses are mean ± standard error of mean, N = 36 using DMRT. * Significant at *p* ≤ 0.05, ** Significant at *p* ≤ 0.01, *** Significant at *p* ≤ 0.001, **** Significant at *p* ≤ 0.0001, ns = Not significant at *p* ≥ 0.05, N = Nitrogen Rates, H = Harvest Time, CoV = Coefficient of Variation, Df = Degree of Freedom, Photo = Photosynthetic Rate, Cond = Stomatal Conductance Rate, TChlC = Total Chlorophyll Content, TCC = Total Carbohydrate Content, TPrC = Total Protein Content, TLiC = Total Lipid Content, TPC = Total Phenolic Content, and TFC = Total Flavonoid Content.

**Table 2:** Effect of N rates and H time on physiology and biochemical assay variables.

	Photo	Cond	TChlC	TCC	TPrC	TLiC	TPC	TFC
N								

N0 (Control)	4.245^a^	0.065^a^	4.782^c^	65.577^d^	32.707^c^	56.556^a^	95.576^b^	11.346^a^
N30	3.432^d^	0.039^c^	5.007^a^	82.452^b^	35.101^b^	49.556^b^	90.473^c^	9.796^b^
N60	3.755^b^	0.053^b^	4.937^b^	187.829^a^	35.874^a^	38.556^d^	100.067^a^	7.608^c^
N90	3.630^c^	0.054^b^	4.910^b^	71.013^c^	28.750^d^	45.000^c^	72.072^d^	5.727^d^

H								

Week 4	8.021^a^	0.084^a^	5.198^a^	61.698^b^	28.709^c^	23.167^c^	68.169^c^	2.546^c^
Week 8	1.863^b^	0.050^b^	4.639^c^	35.980^c^	32.256^b^	39.167^b^	70.976^b^	7.844^b^
Week 12	1.412^c^	0.024^c^	4.889^b^	207.476^a^	38.359^a^	79.917^a^	129.496^a^	15.467^a^

Interaction								

N	<.0001^****^	<.0001^****^	<.0001^****^	<.0001^****^	<.0001^****^	<.0001^****^	<.0001^****^	<.0001^****^
H	<.0001^****^	<.0001^****^	<.0001^****^	<.0001^****^	<.0001^****^	<.0001^****^	<.0001^****^	<.0001^****^
N × H	0.752^ns^	0.268^ns^	<.0001^****^	<.0001^****^	<.0001^****^	<.0001^****^	<.0001^****^	<.0001^****^

*Notes*: All analyses are mean ± standard error of mean, N = 36 using DMRT. * Significant at *p* ≤ 0.05, ** Significant at *p* ≤ 0.01, *** Significant at *p* ≤ 0.001, **** Significant at *p* ≤ 0.0001, ns = Not significant at *p* ≥ 0.05, N = Nitrogen Rates, H = Harvest Time, Photo = Photosynthetic Rate, Cond = Stomatal Conductance Rate, TChlC = Total Chlorophyll Content, TCC = Total Carbohydrate Content, TPrC = Total Protein Content, TLiC = Total Lipid Content, TPC = Total Phenolic Content, and TFC = Total Flavonoid Content.

**Table 3:** Correlation of physiology and biochemical assay variables.

	H	Photo	Cond	TChlC	TCC	TPrC	TLiC	TPC	TFC
H	1.000								
Photo	−0.884****	1.000							
Cond	−0.894****	0.860****	1.000						
TChlC	−0.457***	0.674****		1.000					
TCC	0.597****	0.340*		0.085^ns^	1.000				
TPrC	0.560***	−0.478**		−0.172^ns^	0.542***	1.000			
TLiC	0.792****	−0.563***		−0.331*	0.379*	0.540***	1.000		
TPC	0.596****	−0.394*		−0.059^ns^	0.600****	0.935****	0.667****	1.000	
TFC	0.806****	−0.677****		−0.412*	0.367*	0.632****	0.776****	0.653****	1.000

*Notes*: All analyses are mean ± standard error of mean, N = 36 using DMRT. * Significant at *p* ≤ 0.05, ** Significant at *p* ≤ 0.01, *** Significant at *p* ≤ 0.001, **** Significant at *p* ≤ 0.0001, ns = Not significant at *p* ≥ 0.05, N = Nitrogen Rates, H = Harvest Time, Photo = Photosynthetic Rate, Cond = Stomatal Conductance Rate, TChlC = Total Chlorophyll Content, TCC = Total Carbohydrate Content, TPrC = Total Protein Content, TLiC = Total Lipid Content, TPC = Total Phenolic Content, and TFC = Total Flavonoid Content.
